# Association between Psychosocial Stress and Cardiovascular Disease in Relation to Low Consumption of Fruit and Vegetables in Middle-Aged Men

**DOI:** 10.3390/nu11081915

**Published:** 2019-08-15

**Authors:** Yoonjin Shin, Yangha Kim

**Affiliations:** Department of Nutritional Science and Food Management, Ewha Womans University, Seoul 03760, Korea

**Keywords:** stress, cardiovascular disease, intake of fruit and vegetable, dietary variety score

## Abstract

Stress has been reported to affect dietary intake and chronic disease. This study aimed to investigate the association between psychosocial stress and cardiovascular disease (CVD) in relation to dietary intake. This cross-sectional analysis was performed on 23,792 men enrolled in the Korean Genome and Epidemiology Study from 2004 to 2013. Stress was assessed by the Psychosocial Well-Being Index. Stress level was positively associated with the risks of CVD (odds ratio (OR) for quartile 4 compared to quartile 1 = 1.30 (95% confidence interval 1.24–1.37), *p*-trend < 0.0001), including hypertension (OR = 1.26 (1.20–1.33), *p*-trend < 0.0001), heart disease (OR = 1.55 (1.34–1.80), *p*-trend = 0.0001), and cerebrovascular disease (OR = 2.47 (1.97–3.09), *p*-trend < 0.0001). As the level of stress increased, the intake of fruits and vegetables, as well as antioxidant nutrients, was decreased. Stress level showed an inverse association with dietary variety score (*p*-trend = 0.0001). In addition, dietary variety score was inversely associated with fruits and vegetables consumption (*p*-trend < 0.0001). These results suggest that the CVD risks for those with higher stress levels may be partially related to the decreased consumption of fruits and vegetables and dietary variety score.

## 1. Introduction

Psychosocial stress has been reported to be associated with physical and mental health [1]. While acute stress activates adaptive responses, long-term stress leads to biological changes that weaken stress-related adaptation processes and increase disease susceptibility [1]. Chronic stress stimulation is known to affect the onset of cardiovascular disease (CVD) through excessive cortisol secretion through the hypothalamus pituitary adrenal (HPA) axis [1]. Cortisol may cause an inflammatory reaction, which results in endothelial dysfunction and increased blood pressure, leading to CVD.

CVD is the leading cause of mortality and morbidity in modern societies. About 17.9 million people died from CVD in 2016, accounting for 31% of all global deaths [2]. The most common cause of CVD’s clinical events, such as cerebrovascular diseases and heart disease, is atherosclerotic process [3]. The inflammatory mechanism plays a pivotal role in the mediation of atherosclerosis, from the initial recruitment of circulating leukocytes toward the wall of the arteries, to the rupture of unstable plaques. The risk factors associated with the development of atherosclerotic lesions are influenced by genetic predisposition, but also by environmental factors, particularly diet.

Compelling evidence has indicated that increased consumption of dietary antioxidants might contribute to delayed onset and decreased CVD risk [4]. The most representative antioxidants sources are fruits and vegetables, which can reduce CVD risk through antioxidants that provide vascular defense against oxidative stress by scavenging free radicals and protecting nitric-oxide (NO) from inactivation [5]. In a previous study with the elderly, the highest quintile of fruit or vegetable intake had a 46% lower risk of CVD death compared to those in the lowest quintile [6]. An increase of three servings per day of fruits and vegetables was associated with a significantly lower risk of stroke and cerebrovascular diseases, in a study of 832 men from Framingham [7].

Psychosocial stress has been shown to be associated with an increased risk of CVD [8,9]. Meta-analysis also showed that CVD risk increased by about 50% in relation to high levels of work stress [10]. However, to our knowledge, no previous study has evaluated whether stress status may affect fruit and vegetable consumption, and thereby may relate to the prevalence of CVD. Therefore, we evaluated the association between stress and the risk of CVD in middle-aged Korean men, who participated in the Korean Genome and Epidemiology Study (KoGES). In addition, the relationship between stress status and the consumption of fruit and vegetable was investigated.

## 2. Materials and Methods

### 2.1. Subjects

The KoGES is a consortium project consisting of prospective cohort studies, aimed to develop comprehensive and applicable health care guidelines for common chronic diseases in Koreans [11]. Two subprojects of the KoGES, the health examinee (HEXA) study and the cardiovascular disease association study (CAVAS), survey community-dwellers and participants recruited from the national health examinee registry aged >40 years at baseline. We combined data from the HEXA study (*n* = 173,357) and CAVAS (*n* = 28,338), conducted from 1 January 2004 to 31 December 2013. Based on epidemiological information on the prevalence of cardiovascular risk factors [8,12,13], we restricted our investigation to middle-aged men, who are known to have higher CVD mortality due to stress than women (*n* = 58,043). We excluded participants who reported implausible energy intake (<500 or >4000 kcal/day, *n* = 907), those who did not respond to the stress assessment (*n* = 4201), those who did not know their CVD status (*n* = 6628), and those with a history of diabetes, liver disease, stomach disease, or cancer (*n* = 22,515). The study selected 23,792 men as the final subjects. It was approved by the Institutional Review Board of Ewha Womans University (2019-0041, March 2019), and the procedures followed were in accordance with the Helsinki Declaration of 1975, as revised in 2008.

### 2.2. Assessment of Stress Levels

Stress level was assessed using the Psychosocial Well-being Index—Short Form (PWI-SF) developed by Chang [14], based on the General Health Questionnaire by Goldberg and his colleagues [15]. A feasibility study of PWI-SF has been conducted on Korean laborers and the general public [14]. The PWI-SF consists of 18 items, that are each scored on a four-point Likert scale ranging from ‘strongly disagree’ (0) to ‘strongly agree’ (3). The total score can range from 0 to 54, and a high score indicates high stress. In this study, the subjects were divided into quartiles of score.

### 2.3. Assessment of Dietary Intake

Food intake was collected by well-trained researchers using a semi-quantitative food frequency questionnaire (SQFFQ) that was developed and validated for the KoGES [16]. Subjects were asked to report their average consumption frequency for 106 food items over a period of 12 months (on a nine-point scale of ‘almost none’, ‘once a month’, ‘twice or three times a month’, ‘once or twice a week’, ‘twice or three times a week’, ‘five or six times a week’, ‘once a day’, ‘twice a day’, and ‘three times a day’), as well as their average intake quantity (on a three-point scale of small, medium, and large). The researchers presented pictures of different quantities to the subjects for reference. Definitions of fruits and vegetables subgroups were adapted from a previous study [16]. Additionally, we did not include kimchi or pickled vegetables as vegetables, because of their high salt content. The average daily nutrient intake of each food item was calculated using a weighted frequency per day and a portion size per unit. The nutrient database of the Korean Nutrition Society was used to convert food intake into nutrients [17].

### 2.4. Dietary Variety Score

To evaluate the quality of diet, the dietary variety score (DVS) was adopted and was determined by counting the number of different food items consumed over the course of a day. The method was originally devised by Randall et al. [18]. In this study, we modified the method reported by Choi et al. [19]. Food items that were consumed multiple times during the period were counted only once. Each time another food was added, the DVS was increased by one point. Foods with the same main ingredients, such as pork belly and pork roast, were considered one food. The possible total (*n* = 86) reflected all kinds of food items consumed by the participants in this study.

### 2.5. Classification of CVD

CVD was defined as hypertension, heart disease (angina or myocardial infarction), and cerebrovascular diseases (stroke, cerebral infarction, cerebral hemorrhage, etc.) [20]. Disease histories were collected using an interview-administered questionnaire on any previous diagnosis by a physician and any agents used for treatment.

### 2.6. Assessment of Other Variables

General information on sex, age, education (<high school, ≥high school), monthly income (<2 million KRW, ≥2 million KRW), current alcohol drinker (yes, no), current smoking status (yes, no), and moderate-intensity physical activity (yes, no) was collected using an interview-administered questionnaire. The height and weight of the subjects were measured under the condition that each subject took off their shoes and clothes and wore only a patient gown. Body mass index (BMI) was calculated as weight (kg)/height (m^2^). The circumference of the waist (cm) was measured by a non-stretchable standard tape at the thinnest part of the waist between the ribs and long bone. Each measurement was performed three times to calculate the average values. The blood pressure was measured two times in the right arm using an automatic sphygmomanometer or standardized mercury, depending on the institution. All subjects underwent blood sample collection after having fasted for more than 8 h. The collected blood samples were centrifuged on site, and then high-sensitive C-reactive protein (hs-CRP), total cholesterol (TC), high-density lipoprotein cholesterol (HDL-C), and triglycerides (TG) were analyzed using the enzyme method (ADVIA 1650 and ADVIA 1800; Siemens Healthineers, Deerfield, IL, USA). Low-density lipoprotein cholesterol (LDL-C) was calculated by the following equation described by Friedewald with TG concentrations <400 mg/dL [21]: LDL-C = [TC (mg/dL) – {HDL-C (mg/dL) – (TG (mg/dL)/5)}].

### 2.7. Statistical Analysis

Subjects were categorized into quartiles for the relative comparison of stress levels according to PWI-score. Data were expressed as the mean ± standard error (SE) for continuous variables and as percentages for categorical variables. The general linear model and the Cochran–Mantel–Haenszel analysis were used to determine differences in the means and distribution of general characteristics and to test the linear trends according to stress level. Adjustments were performed for potential confounding variables, that were either statistically significant in univariate analyses or known to be potentially important factors related to dietary intake and stress-induced chronic disease, such as age, BMI, exercise, alcohol drinking, smoking, education, and income. Multivariate logistic regression analysis was applied to obtain odds ratios (ORs) and corresponding 95% confidence intervals (CIs) for the risk of CVD. All statistical analyses were performed using SAS v9.4 (SAS Institute Inc., Cary, NC, USA), and *p*-values of 0.05 were considered significant.

## 3. Results

Table 1 presents general characteristics according to the quartile of stress level. The median PWI-SF score ranged from 7.0 in the lowest quartile to 23.0 in the highest quartile. Subjects with higher stress levels tended to be slightly younger, more likely to smoke, less likely to exercise, less educated, earning less income, and showing a smaller waist circumference and lower BMI compared with those with lower stress levels. In addition, subjects with higher stress levels showed higher concentrations of hs-CRP and TG compared with those with lower stress levels.

The ORs and 95% CIs of CVD according to quartile of stress level are presented Table 2. After adjustment for age, BMI, alcohol drinking, smoking, exercise, education level, and income status, we observed a significant positive association between the stress level and OR of CVD. Quartile 4 of stress level compared with Quartile 1 was associated with a higher OR of CVD (OR: 1.38; 95% CI: 1.26–1.51; *p*-trend < 0.0001), hypertension (1.31; 1.19–1.43; *p*-trend < 0.0001), heart disease (1.52; 1.17–1.97; *p*-trend = 0.002), and cerebrovascular disease (2.80; 1.90–4.14; *p*-trend < 0.0001).

Dietary intake classified by stress level is shown in Table 3. As the level of stress increased, the consumption of fruits and vegetables decreased. The intake of antioxidant nutrients, such as vitamin A, vitamin C, vitamin E, iron, and zinc, also decreased. In addition, subjects with higher stress levels showed a lower DVS, reflecting greater dietary diversity than subjects with lower stress levels. There was a significant relationship between dietary variety and the consumption of fruits and vegetables (Table 4). As the DVS decreased, the consumption of fruits and vegetables decreased.

## 4. Discussion

This study investigated the association between psychosocial stress and CVD in relation to dietary intake. Subjects with higher stress levels showed a higher risk of CVD compared to those with lower stress levels. Stress level was inversely associated with the intake of antioxidant nutrients, fruit, and vegetables. In addition, subjects with higher stress levels showed lower dietary variety. There were also declining trends in the consumption of fruit and vegetables with lower dietary variety.

In this study, subjects with higher stress levels showed higher serum hs-CRP concentrations than those with lower stress levels. The hs-CRP is an inflammatory marker synthesized by hepatocytes and is related to a decrease in NO concentration due to reduced endothelial NO synthase activity [22]. This reduction may increase the risk of endothelial dysfunction by reducing endothelium-dependent vasodilation [23]. Thus, changes in stress-induced procoagulant could be associated with biological pathways for CVD. Increased CRP associated with behavioral stress has been demonstrated in healthy men and patients with coronary artery disease [24]. Greenwood et al. [25] reviewed 14 prospective studies on humans, and almost all studies found a positive relationship between stress and CVD. In a study involving attorneys, psychological stress, such as that resulting from a high work load, was reported to be associated with the development of coronary artery disease [26]. These findings are similar to our finding that stress level showed a significant association with the OR of CVD. If all of these findings are taken into consideration, it can be suggested that there is a positive association between stress and the risk of CVD.

Our subjects with higher stress levels showed a lower intake of fruit and vegetables, as well as antioxidant nutrients. Stress has been reported to induce the activation of sympathetic adrenal medullary systems, with the release of adrenaline and noradrenaline. These hormones may suppress the appetite [27]. It can be assumed that as appetite decreases, the amount and number of consumed foods decrease. Also, the reduction in the number of foods consumed is linked to a decrease in dietary variety. According to a previous study [28], perceived stress showed a relationship with a low-quality diet and low dietary variety. In addition, lower dietary variety has been associated with a low intake of healthy foods, such as vegetables and fruits [29]. Among Iranian females, a diet low in variety was related to lower fruit and vegetable consumption [30]. In this study, consistent with these results, there was a declining trend in the consumption of fruits and vegetables with lower dietary variety.

Fruit and vegetable consumption has been reported to reduce CVD risk through beneficial combinations of various antioxidants [5]. In particular, antioxidant vitamins, such as vitamin A, vitamin C, and vitamin E have received the most attention with regard to CVD prevention. Higher intakes of these vitamins were associated with a reduced risk of stroke or coronary heart disease in several observational investigations [31,32,33]. According to the oxidation hypothesis of atherosclerosis, the improvement of early atherogenic lesions depends on antioxidant defense systems, including diet-induced compounds such as antioxidant nutrients, and these improve endothelial function by reducing LDL oxidation [34]. The American Heart Association recommends more than five servings of fruit and vegetables daily to prevent CVD [35]. Our subjects with higher stress levels showed a lower consumption of fruits and vegetables than those with lower stress levels. Thus, the low consumption of fruits and vegetables in subjects with higher stress levels might contribute to a higher risk of CVD.

The associations observed in this study could also be due to the fact that having CVD leads to stress and reduced fruit and vegetable consumption. Stress is not only a risk factor for CVD, it is also common after cardiovascular events. More than half of the people suffering from a stroke experience stress symptoms within 18 months of the stroke [36]. In addition, about one in six patients with myocardial infarction experience mental health deprivation, and at least twice that many suffer from severe depressive symptoms [37]. Thus, CVD may induce stress and reduce fruit and vegetable consumption.

There are several limitations to this study. First, whether stress is associated with fruit and vegetable consumption and CVD was analyzed by an observational cross-sectional study design, and causality cannot be confirmed. Second, dietary intake was analyzed through the SQFFQ, which may be an inaccurate representation of real consumption amounts. Well-trained interviewers with a validated SQFFQ and pictures of portion sizes performed the survey, but measurement errors in dietary intake were inevitable. Despite these limitations, this seems to be the first study to examine the association between psychosocial stress and CVD in relation to fruit and vegetable consumption.

## 5. Conclusions

This study used data from the KoGES to demonstrate that stress is positively associated with the OR of CVD in middle-aged Korean men. Subjects with higher stress levels showed a lower dietary variety score. In addition, there was a declining trend in the consumption of fruits and vegetables with a lower dietary variety score. Therefore, it can be suggested that the high CVD risk for those with higher stress levels is partially related to a decreased consumption of fruits and vegetables and dietary variety score.

## Figures and Tables

**Table 1:** General characteristics of the participants according to quartile of stress level.

	Stress Level ^§^				*p*-Trend
	Quartile 1 (*n* = 4846)	Quartile 2 (*n* = 7470)	Quartile 3 (*n* = 5270)	Quartile 4 (*n* = 6206)	
PWI-SF score (median)	7.0	11.0	16.0	23.0	
Age (years)	51.7 (0.1)	51.2 (0.1)	50.9 (0.1)	50.8 (0.1)	
Height (cm)	168.8 (0.1)	169.2 (0.1)	169.1 (0.1)	168.8 (0.1)	0.067
Weight (kg)	70.2 (0.1)	70.1 (0.1)	69.6 (0.1)	69.1 (0.1)	<0.0001
Waist circumference (cm)	85.9 (0.1)	85.5 (0.1)	85.4 (0.1)	85.2 (0.1)	<0.0001
Body mass index (kg/m^2^)	24.6 (0.04)	24.4 (0.03)	24.3 (0.04)	24.2 (0.04)	<0.0001
Systolic blood pressure (mmHg)	126.0 (0.2)	125.4 (0.2)	125.2 (0.2)	125.2 (0.2)	0.012
Diastolic blood pressure (mmHg)	79.1 (0.2)	78.6 (0.1)	78.8 (0.1)	79.2 (0.1)	0.312
hs-CRP (mg/dL)	1.42 (0.04)	1.65 (0.05)	1.63 (0.06)	1.72 (0.05)	0.0002
Total cholesterol (mg/dL)	194.8 (0.5)	196.7 (0.4)	196.2 (0.5)	194.8 (0.4)	0.645
HDL cholesterol (mg/dL)	50.3 (0.2)	50.0 (0.1)	49.6 (0.2)	50.1 (0.2)	0.121
LDL cholesterol (mg/dL)	116.2 (0.5)	118.5 (0.4)	117.5 (0.4)	115.4 (0.4)	0.061
Triglycerides (mg/dL)	149.6 (1.6)	147.1 (1.1)	151.9 (1.5)	154.4 (1.4)	0.009
Total cholesterol/HDL cholesterol	4.05 (0.02)	4.12 (0.01)	4.14 (0.02)	4.08 (0.02)	0.091
Triglycerides/HDL cholesterol	3.33 (0.05)	3.28 (0.03)	3.44 (0.05)	3.46 (0.05)	0.013
Current alcohol drinker (%)	73.4	76.5	76.2	75.2	0.440
Current smoker (%)	29.6	33.6	36.2	40.6	<0.0001
Moderate exercise activity (%)	61.0	55.8	50.9	44.3	<0.0001
Education (≧high school, %)	78.3	81.6	79.4	73.8	<0.0001
Monthly income (≧2 million KRW, %)	79.7	80.8	77.7	71.6	<0.0001

hs-CRP, high-sensitive C-reactive protein; HDL, high-density lipoprotein; LDL, low-density lipoprotein; KRW, Korean won. Values are expressed as mean (SE) or percentage. The *p*-trend was obtained in a general linear model analysis and Cochran–Mantel–Haenszel analysis with adjustment for age. ^§^ Stress level was assessed using the Psychosocial Well-being Index–Short Form (PWI-SF) developed by Chang [14].

**Table 2:** Odds ratios (95% confidence intervals) for cardiovascular disease of participants according to quartile of stress level.

	Stress Level ^§^				*p*-Trend
	Quartile 1 (*n* = 4846)	Quartile 2 (*n* = 7470)	Quartile 3 (*n* = 5270)	Quartile 4 (*n* = 6206)	
Cardiovascular disease					
Number of cases	1608	2348	1750	2267	
Model 1 ^†^	1.0	0.98 (0.90–1.06)	1.12 (1.03–1.22)	1.34 (1.23–1.46)	<0.0001
Model 2 ^‡^	1.0	0.98 (0.90–1.07)	1.12 (1.02–1.23)	1.38 (1.26–1.51)	<0.0001
Hypertension					
Number of cases	1523	2211	1659	2100	
Model 1 ^†^	1.0	0.97 (0.89–1.05)	1.12 (1.02–1.22)	1.28 (1.18–1.39)	<0.0001
Model 2 ^‡^	1.0	0.97 (0.89–1.06)	1.11 (1.01–1.22)	1.31 (1.19–1.43)	<0.0001
Heart disease					
Number of cases	119	179	131	195	
Model 1 ^†^	1.0	1.04 (0.82–1.31)	1.12 (0.87–1.45)	1.45 (1.15–1.83)	0.003
Model 2 ^‡^	1.0	1.13 (0.88–1.46)	1.15 (0.87–1.52)	1.52 (1.17–1.97)	0.002
Cerebrovascular disease					
Number of cases	46	78	64	138	
Model 1 ^†^	1.0	1.16 (0.81–1.68)	1.41 (0.96–2.07)	2.60 (1.86–3.65)	<0.0001
Model 2 ^‡^	1.0	1.36 (0.91–2.05)	1.46 (0.94–2.26)	2.80 (1.90–4.14)	<0.0001

OR, odds ratio; CI, confidence interval. ORs (95% CIs) were determined by the logistic regression model. ^§^ Stress level was assessed using the Psychosocial Well-being Index–Short Form (PWI-SF) developed by Chang [14]. ^†^ Model 1 was adjusted for age and body mass index. ^‡^ Model 2 was adjusted for age, body mass index, alcohol intake, smoking, exercise, education level, and income status.

**Table 3:** Dietary intake status of the participants according to quartile of stress level.

	Stress Level ^§^				*p*-Trend
	Quartile 1 (*n* = 4846)	Quartile 2 (*n* = 7470)	Quartile 3 (*n* = 5270)	Quartile 4 (*n* = 6206)	
Fruits and vegetables (g)	159.0 (1.6)	147.7 (1.1)	138.2 (1.3)	127.4 (1.2)	<0.0001
Fruits (g)	97.6 (1.3)	90.2 (0.9)	83.0 (1.1)	73.0 (1.0)	<0.0001
Vegetables (g)	61.4 (0.7)	57.5 (0.5)	55.2 (0.6)	54.4 (0.6)	<0.0001
Energy (kcal)	1891.7 (7.4)	1862.7 (5.4)	1839.7 (6.9)	1807.7 (6.7)	<0.0001
Carbohydrate (g)	177.3 (0.2)	175.7 (0.2)	177.9 (0.2)	177.8 (0.2)	0.0002
Protein (g)	33.7 (0.1)	34.1 (0.1)	33.0 (0.1)	32.8 (0.1)	<0.0001
Fat (g)	16.03 (0.08)	16.60 (0.07)	15.92 (0.08)	15.97 (0.08)	0.002
Vitamin A (R.E.)	274.6 (2.3)	257.6 (1.6)	249.2 (1.9)	245.9 (1.9)	<0.0001
Vitamin C (mg)	56.1 (0.4)	53.3 (0.3)	50.8 (0.4)	47.9 (0.3)	<0.0001
Vitamin E (mg)	4.5 (0.02)	4.4 (0.02)	4.3 (0.02)	4.2 (0.02)	<0.0001
Fe (mg)	5.5 (0.02)	5.4 (0.02)	5.3 (0.02)	5.2 (0.02)	<0.0001
Zinc (ug)	4.5 (0.02)	4.6 (0.01)	4.4 (0.01)	4.4 (0.01)	<0.0001
Fiber (g)	3.23 (0.02)	3.05 (0.01)	3.00 (0.02)	2.93 (0.01)	<0.0001
Dietary variety score ^†^	59.7 (0.2)	60.4 (0.1)	59.7 (0.2)	58.0 (0.2)	0.0001

Values were expressed as mean (SE). Food and nutrient intake was represented as grams per 1000 kcal. The *p*-trend was obtained through general linear model analysis with adjustment for age, body mass index, alcohol intake, smoking, exercise, education level, and income status. ^§^ Stress level was assessed using the Psychosocial Well-being Index—Short Form (PWI-SF) developed by Chang [14]. ^†^ Dietary variety score was determined by counting the number of different food items consumed over the course of a day [19].

**Table 4:** Fruit and vegetable consumption according to quartile of dietary variety score.

	Dietary Variety Score ^§^				*p*-Trend
	Quartile 1 (*n* = 5865)	Quartile 2 (*n* = 5959)	Quartile 3 (*n* = 5928)	Quartile 4 (*n* = 6040)	
Median (score)	44.0	56.0	65.0	74.0	
Fruits and vegetables	116.2 (1.3)	147.7 (1.3)	156.0 (1.3)	150.1 (1.2)	<0.0001
Fruits	70.4 (1.1)	90.5 (1.1)	93.6 (1.0)	87.9 (1.0)	<0.0001
Vegetables	45.8 (0.6)	57.2 (0.6)	62.4 (0.6)	62.2 (0.6)	<0.0001

Values were expressed as the mean (SE). Food and nutrient intake was represented as grams per 1000 kcal. The *p*-trend was obtained through general linear model analysis with adjustment for age, body mass index, alcohol intake, smoking, exercise, education level, and income status. ^§^ Dietary variety score was determined by counting the number of different food items consumed over the course of a day [19].
